# Supplementary material for: Metabolic Responses, Cell Recoverability, and Protein Signatures of Three Extremophiles: Sustained Life During Long-Term Subzero Incubations
Source: Microorganisms. 2025 Jan 24;13(2):251. doi: 10.3390/microorganisms13020251 (PMC11858272; doi:10.3390/microorganisms13020251)
Supplement: Supplementary file 1 [file microorganisms-13-00251-s001.zip › Chart S1.pdf]

Chart S1. Experiment timeline

Experiment Phase	Starting	Ending	Strain
**PHASE 1**	March 25, 2019	March 24, 2020	*Colwellia psychrerythraea* str. 34H
**PHASE 2**	May 15, 2019	May 15, 2020	*Psychrobacter sp.* str 7E
**PHASE 3**	July 22, 2019	July 23, 2020	*Halobacter sp.* str 3E

JANUARY

M	T	W	T	F	S	S

FEBRUARY

M	T	W	T	F	S	S

MARCH

M	T	W	T	F	S	S
				1	2	3
4	5	6	7	8	9	10
11	12	13	14	15	16	17
18	19	20	21	22	23	24
25	26	27	28	29	30	31

APRIL

M	T	W	T	F	S	S
1	2	3	4	5	6	7
8	9	10	11	12	13	14
15	16	17	18	19	20	21
22	23	24	25	26	27	28
29	30					

MAY

M	T	W	T	F	S	S
		1	2	3	4	5
6	7	8	9	10	11	12
13	14	15	16	17	18	19
20	21	22	23	24	25	26
27	28	29	30	31		

JUNE

M	T	W	T	F	S	S
					1	2
3	4	5	6	7	8	9
10	11	12	13	14	15	16
17	18	19	20	21	22	23
24	25	26	27	28	29	30

JULY

M	T	W	T	F	S	S
1	2	3	4	5	6	7
8	9	10	11	12	13	14
15	16	17	18	19	20	21
22	23	24	25	26	27	28
29	30	31				

AUGUST

M	T	W	T	F	S	S
			1	2	3	4
5	6	7	8	9	10	11
12	13*	14	15	16	17	18
19	20	21	22	23	24	25
26	27	28	29	30	31	

SEPTEMBER

M	T	W	T	F	S	S
						1
2	3	4	5	6	7	8
9	10	11	12	13	14	15
16	17	18	19	20	21	22
23	24	25	26	27	28	29
30						

OCTOBER

M	T	W	T	F	S	S
	1	2	3	4	5	6
7	8	9	10	11	12	13
14	15	16	17	18	19	20
21	22	23	24	25	26	27
28	29	30	31			

NOVEMBER

M	T	W	T	F	S	S
				1	2	3
4	5	6	7	8	9	10
11	12	13	14	15	16	17
18	19	20	21	22	23	24
25	26	27	28	29	30	

DECEMBER

M	T	W	T	F	S	S
						1
2	3	4	5	6	7	8
9	10	11	12	13	14	15
16	17	18	19	20	21	22
23	24	25	26	27	28	29
30	31					

1/2

Day	Samples collected (in triplicate)
Experiment set-up– all samples are placed in incubation<br>(March 25, May 15, Jul 22)	3H-Leu incorporation: 0, 1, 2, 4, 8, 12, 24 h<br>Protein-SIP: 0, 1, 2, 4, 8, 12, 24 h<br>Cell numbers: 0, 24 h<br>Recoverability: 0, 24 h
7 d after set-up	3H-Leu incorporation<br>Protein-SIP<br>Cell numbers<br>Recoverability
14 d, 1 mo, 2 mo, 6 mo and 12 mo after set-up	3H-Leu incorporation<br>Cell numbers<br>Recoverability

*H3E 14 d measurement occurred at 21 d

Experiment Phase	Starting	Ending	Strain
**PHASE 1**	March 25, 2019	March 24, 2020	*Colwellia psychrerythraea* str. 34H
**PHASE 2**	Apr 22, 2019	Apr 22, 2020	*Psychrobacter sp.* str 7E
**PHASE 3**	July 22, 2019	July 23, 2019	Halobacter sp. str 3E

JANUARY

M	T	W	T	F	S	S
		1	2	3	4	5
6	7	8	9	10	11	12
13	14	15	16	17	18	19
20	21	22	23	24	25	26
27	28	29	30	31		

FEBRUARY

M	T	W	T	F	S	S
					1	2
3	4	5	6	7	8	9
10	11	12	13	14	15	16
17	18	19	20	21	22	23
24	25	26	27	28		

MARCH

M	T	W	T	F	S	S
					1	2
3	4	5	6	7	8	9
10	11	12	13	14	15	16
17	18	19	20	21	22	23
24	25	26	27	28	29	30
31						

APRIL

M	T	W	T	F	S	S
	1	2	3	4	5	6
7	8	9	10	11	12	13
14	15	16	17	18	19	20
21	22	23	24	25	26	27
28	29	30				

MAY

M	T	W	T	F	S	S
			1	2	3	4
5	6	7	8	9	10	11
12	13	14	15	16	17	18
19	20	21	22	23	24	25
26	27	28	29	30	31	

JUNE

M	T	W	T	F	S	S
						1
2	3	4	5	6	7	8
9	10	11	12	13	14	15
16	17	18	19	20	21	22
23	24	25	26	27	28	29
30						

JULY

M	T	W	T	F	S	S
	1	2	3	4	5	6
7	8	9	10	11	12	13
14	15	16	17	18	19	20
21	22	23	24	25	26	27
28	29	30	31			

AUGUST

SEPTEMBER

OCTOBER

NOVEMBER

DECEMBER
