# Supplementary material for: Metabolic Responses, Cell Recoverability, and Protein Signatures of Three Extremophiles: Sustained Life During Long-Term Subzero Incubations
Source: Microorganisms. 2025 Jan 24;13(2):251. doi: 10.3390/microorganisms13020251 (PMC11858272; doi:10.3390/microorganisms13020251)
Supplement: Supplementary file 1 [file microorganisms-13-00251-s001.zip › Table S1.pdf]

Table S1. Known Temperature and Salinity Growth Ranges for P7E and H3E. Microorganisms were cultured aseptically in half-strength Marine Broth 2216 (Difco laboratories, Detroit, MI), 3 or 4 replicas, not mixing. Growth was visually assessed by turbidity, and for *Psychrobacter sp.* strain 7E with additional microscopic observations. "Yes" indicates turbidity in all replicas. Shaded areas of the table: growth not assessed. Data combined from experiments performed by M. Ewert, J. Schermer and S. Carpenter at the University of Washington, School of Oceanography, at the J.W. Deming lab, during 2007-2014

Isolate	Temp. (°C)	Salinity (ppt)							
		17	22	27	35	65	98	125	220
Psychrobacter sp. str. 7E	−14	No†	No	No	No				
	−8	Yes	Yes	Yes	Yes				
	−1	Yes	Yes	Yes	Yes (1 week)	Yes	Yes	Yes	No
	2				Yes (1 week)				
	8				Yes				
	25				Yes (overnight)				
Halomonas sp. str. 3E	−1				Yes	Yes	Yes	Yes	Yes
	25				Yes (overnight)				

†Two out of four replicates showed microscopic counts of 2.26×10^6 cells/mL
