# Supplementary material for: Metabolic Responses, Cell Recoverability, and Protein Signatures of Three Extremophiles: Sustained Life During Long-Term Subzero Incubations
Source: Microorganisms. 2025 Jan 24;13(2):251. doi: 10.3390/microorganisms13020251 (PMC11858272; doi:10.3390/microorganisms13020251)
Supplement: Supplementary file 1 [file microorganisms-13-00251-s001.zip › Table S2.pdf]

Table S2. Generation Time at Low Temperatures.

Organism	Temp. (°C)	Generation time (h)	Media	State	Citation	Notes
Chryseobacterium sp. V3519-10	-5	1040	M9 + 5.7% NaCl	Liquid	[14]	Values provided in Table 1, converted from CTC-live cell counts
Chryseobacterium sp. V3519-10	-5	1040	M9 mineral media	Liquid	[14]	Converted from values calculated in Table 1 w/ factor of 0.693*
Clostridium algoriphilum sp. nov.	-2	50	Methods	Unclear	[80]	Data from digitized plot
Clostridium algoriphilum sp. nov.	0	29	Methods	Unclear	[80]	Data from digitized plot
Clostridium algoriphilum sp. nov.	3	19.13	Methods	Unclear	[80]	Data from digitized plot
Clostridium algoriphilum sp. nov.	6	15.02	Methods	Unclear	[80]	Data from digitized plot
Clostridium algoriphilum sp. nov.	12	28.1	Methods	Unclear	[80]	Data from digitized plot
Clostridium algoriphilum sp. nov.	15	46	Methods	Unclear	[80]	Data from digitized plot
Colwellia psychrerythraea 34H	-5	1903	ASW-	Liquid	[37]	Converted w/ factor of 0.693
Colwellia psychrerythraea 34H	-5	29.21	ASW+Glu/YE	Liquid	[37]	Converted w/ factor of 0.693
Colwellia psychrerythraea 34H	-5	92225	Brine-	Liquid	[37]	Converted w/ factor of 0.693
Colwellia psychrerythraea 34H	-5	1471	Brine+Glu/YE	Liquid	[37]	Converted w/ factor of 0.693
Colwellia psychrerythraea	-10	1064	ASW-	Liquid	[37]	Converted w/ factor of 0.693

Organism	Temp. (°C)	Generation time (h)	Media	State	Citation	Notes
34H						
Colwellia psychrerythraea 34H	-10	43.62	ASW+Glu/YE	Liquid	[37]	Converted w/ factor of 0.693
Colwellia psychrerythraea 34H	-10	1907560	Brine-	Liquid	[37]	Converted w/ factor of 0.693
Colwellia psychrerythraea 34H	-10	4717	Brine+Glu/YE	Liquid	[37]	Converted w/ factor of 0.693
Colwellia psychrerythraea 34H (2015)	-1	245	ASW+GTLV+EPS	Liquid	†	
Colwellia psychrerythraea 34H (2015)	-5	1031	ASW+GTLV+EPS	Liquid?	†	
Colwellia psychrerythraea 34H (2015)	-10	9700	ASW+GTLV+EPS	Solid?	†	
Colwellia psychrerythraea 34H (2015)	-20	3162545	ASW+GTLV+EPS	Solid	†	
Colwellia psychrerythraea 34H (2015)	-196	18393762	ASW+GTLV+EPS	Solid	†	
Colwellia psychrerythraea 34H (Huston)	-5.9	98.4	5mL 2216 (full strength)	Liquid?	[35], as cited in [49]	Note: Doesn't match data in [35] (e.g. Table 4.3)
Colwellia psychrerythraea 34H (Huston)	-4.5	103	5mL 2216 (full strength)	Liquid?	[35]	5mL version
Colwellia psychrerythraea 34H (Huston)	-3	98	5mL 2216 (full strength)	Liquid	[35]	5mL version
Colwellia psychrerythraea 34H (Huston)	-1.8	86	5mL 2216 (full strength)	Liquid	[35]	5mL version
Colwellia psychrerythraea 34H (Huston)	-0.5	76	5mL 2216 (full strength)	Liquid	[35]	5mL version

Organism	Temp. (°C)	Generation time (h)	Media	State	Citation	Notes
Colwellia psychrerythraea 34H (Huston)	0.9	75	5mL 2216 (full strength)	Liquid	[35]	5mL version
Colwellia psychrerythraea 34H (Huston)	2.2	56	5mL 2216 (full strength)	Liquid	[35]	5mL version
Colwellia psychrerythraea 34H (Huston)	3.7	52	5mL 2216 (full strength)	Liquid	[35]	5mL version
Colwellia psychrerythraea 34H (Huston)	4.9	34	5mL 2216 (full strength)	Liquid	[35]	5mL version
Colwellia psychrerythraea 34H (Huston)	6.4	30	5mL 2216 (full strength)	Liquid	[35]	5mL version
Colwellia psychrerythraea 34H (Huston)	7.6	28	5mL 2216 (full strength)	Liquid	[35]	5mL version
Colwellia psychrerythraea 34H (Huston)	9.1	24	5mL 2216 (full strength)	Liquid	[35]	5mL version
Colwellia psychrerythraea 34H (Huston)	10.4	32	5mL 2216 (full strength)	Liquid	[35]	5mL version
Colwellia psychrerythraea 34H (Huston)	11.8	34	5mL 2216 (full strength)	Liquid	[35]	5mL version
Colwellia psychrerythraea 34H (Huston)	13.1	33	5mL 2216 (full strength)	Liquid	[35]	5mL version
Colwellia psychrerythraea 34H (Huston)	14.3	45	5mL 2216 (full strength)	Liquid	[35]	5mL version
Colwellia psychrerythraea 34H (Huston)	15.8	55	5mL 2216 (full strength)	Liquid	[35]	5mL version
Colwellia psychrerythraea 34H (Huston)	17	82	5mL 2216 (full strength)	Liquid	[35]	5mL version
Colwellia psychrerythraea	18.5	278	5mL 2216 (full	Liquid	[35]	5mL version

Organism	Temp. (°C)	Generation time (h)	Media	State	Citation	Notes
34H (Huston)			strength)			
Colwellia psychrerythraea 34H (Huston)	-1	48	80mL 2216 (full strength)	Liquid	[35]	80mL version
Colwellia psychrerythraea 34H (Huston)	-1	10	500mL 2216, shaken (full strength)	Liquid	[35]	500mL + shaken (100rpm) version
Colwellia psychrerythraea 34H (Huston)	-1	6	1000mL 2216, stirred (full strength)	Liquid	[35]	1000mL + stirred (140rpm) version
Colwellia psychrerythraea 34H (Huston)	4	30	80mL 2216 (full strength)	Liquid	[35]	80mL version
Colwellia psychrerythraea 34H (Huston)	8	17	80mL 2216 (full strength)	Liquid	[35]	80mL version
Colwellia psychrerythraea 34H (Huston)	9.1	4	500mL 2216, shaken (full strength)	Liquid	[35]	500mL + shaken (100rpm) version
Colwellia psychrerythraea 34H (Huston)	9.1	3	1000mL 2216, stirred (full strength)	Liquid	[35]	1000mL + stirred (140rpm) version
Colwellia psychrerythraea 34H (Huston)	13.1	20	80mL 2216 (full strength)	Liquid	[35]	80mL version
Colwellia psychrerythraea 34H (Huston)	18	111	80mL 2216 (full strength)	Liquid	[35]	80mL version
Colwellia psychrerythraea 34H (Phase 1)	8	3115	ASW+GTLV	Liquid	‡	
Colwellia psychrerythraea 34H (Phase 1)	-5	6459	ASW+GTLV	Liquid	‡	
Colwellia psychrerythraea	-10	355388	ASW+GTLV	Solid	‡	

Organism	Temp. (°C)	Generation time (h)	Media	State	Citation	Notes
34H (Phase 1)						
Colwellia psychrerythraea 34H (Phase 1)	-36	295122	ASW+GTLV	Solid	‡	
Exiguobacterium sp. strain 255-15	-2.5	132	TSB	Liquid	[79]	Values stated in text; data can be extracted from Fig 1 for temps btwn -0.5C to 42C
Frigoribacterium aff. faeni A-1/C-an/E	-10	4892		Liquid	[77]	Specific growth rate converted w factor of 0.693
Halomonas sp. 3E	22	291	ASW+GTLV+EPS	Liquid	†	
Halomonas sp. 3E	-5	2074	ASW+GTLV+EPS	Liquid	†	
Halomonas sp. 3E	-36	30334	ASW+GTLV+EPS	Solid	†	
Paenisporosarcina sp. Eur1 9.01.10	25	11.04	TSB	Liquid	[78]	Values in Table S4
Paenisporosarcina sp. Eur1 9.01.10	25	41.52	TSB+5%salt+5%sucrose	Liquid	[78]	Values in Table S4
Paenisporosarcina sp. Eur1 9.01.10	5	108	TSB+5%salt+5%sucrose	Liquid	[78]	Values in Table S4
Paenisporosarcina sp. Eur1 9.01.10	-5	350.4	TSB+5%salt+5%sucrose	Liquid	[78]	Values in Table S4
Planococcus halocryophilus Or1	37	9.11	TSB	Liquid	[16]	Data from digitized plot
Planococcus halocryophilus Or1	30	7.56	TSB	Liquid	[16]	Data from digitized plot
Planococcus halocryophilus Or1	25	6.75	TSB	Liquid	[16]	Data from digitized plot

Organism	Temp. (°C)	Generation time (h)	Media	State	Citation	Notes
Planococcus halocryophilus Or1	15	13.37	TSB	Liquid	[16]	Data from digitized plot
Planococcus halocryophilus Or1	20	10	TSB	Liquid	[16]	Data from digitized plot
Planococcus halocryophilus Or1	15	17.87	TSB	Liquid	[16]	Data from digitized plot
Planococcus halocryophilus Or1	10	44.03	TSB	Liquid	[16]	Data from digitized plot
Planococcus halocryophilus Or1	5	272.78	TSB	Liquid	[16]	Data from digitized plot
Planococcus halocryophilus Or1	-5	332.14	TSB-cryo	Liquid	[16]	Data from digitized plot
Planococcus halocryophilus Or1	-10	960	TSB-cryo	Liquid	[16]	Data reported in paper
Planococcus halocryophilus Or1	-15	1200	TSB-cryo	Liquid	[16]	Data reported in paper
Polaromonas sp. Eur3 1.2.1	22	81.6	R2A	Liquid	[78]	Values in Table S4
Polaromonas sp. Eur3 1.2.1	10	74.4	R2A	Liquid	[78]	Values in Table S4
Polaromonas sp. Eur3 1.2.1	0	86.4	R2A	Liquid	[78]	Values in Table S4
Psychrobacter cryopegalla	-10	894.48	R2A + 3% NaCl	Unclear	[81]	Data from digitized plot
Psychrobacter cryopegalla	-4	147.67	R2A + 3% NaCl	Unclear	[81]	Data from digitized plot
Psychrobacter cryopegalla	-2	89.93	R2A + 3% NaCl	Unclear	[81]	Data from digitized plot
Psychrobacter cryopegalla	0	42.26	R2A + 3% NaCl	Unclear	[81]	Data from digitized plot
Psychrobacter cryopegalla	2	38.81	R2A + 3% NaCl	Unclear	[81]	Data from digitized plot
Psychrobacter cryopegalla	4	19.46	R2A + 3% NaCl	Unclear	[81]	Data from digitized plot
Psychrobacter cryopegalla	7	14.4	R2A + 3% NaCl	Unclear	[81]	Data from digitized plot

Organism	Temp. (°C)	Generation time (h)	Media	State	Citation	Notes
Psychrobacter cryopegalla	10	10.21	R2A + 3% NaCl	Unclear	[81]	Data from digitized plot
Psychrobacter cryopegalla	16	3.79	R2A + 3% NaCl	Unclear	[81]	Data from digitized plot
Psychrobacter cryopegalla	22	2.72	R2A + 3% NaCl	Unclear	[81]	Data from digitized plot
Psychrobacter sp. 7E	22	628	ASW+GTLV+EPS	Liquid	†	
Psychrobacter sp. 7E	-5	1549	ASW+GTLV+EPS	Liquid	†	
Psychrobacter sp. 7E	-10	76302	ASW+GTLV+EPS	Solid	†	
Psychrobacter sp. 7E	-36	633716	ASW+GTLV+EPS	Solid	†	
Psychrobacter sp. PAMC 21119	20	2.4	Marine Broth	Unclear	[76]	data listed in fig 1
Psychrobacter sp. PAMC 21119	5	5.5	Marine Broth	Unclear	[76]	data listed in fig 1
Psychrobacter sp. PAMC 21119	0	16.4	Marine Broth	Unclear	[76]	data listed in fig 1
Psychrobacter sp. PAMC 21119	-5	27.4	Marine Broth	Unclear	[76]	data listed in fig 1
Psychrobacter sp. St1	-10	1040	R2A+NaCl	Liquid	[77]	Specific growth rate converted w factor of 0.693
Psychrobacter sp. strain 215-51	-2.5	84	TSB	Liquid	[79]	Values stated in text; data can be extracted from Fig 1 for temps btwn -0.5C to 28C
Psychrobacter sp. strain 273-4	-2.5	84	TSB	Liquid	[79]	Values stated in text; data can be extracted from Fig 1 for temps btwn -0.5C to 28C

Organism	Temp. (°C)	Generation time (h)	Media	State	Citation	Notes
Psychromonas ingrahamii	-12	240	Ordal's SWC-m + glycerol (50g/L)	Liquid	[74]	Suspected to be capable of growth at -15C, but unable to keep media liquid
Psychromonas ingrahamii	5	12	Ordal's SWC-m + glycerol (50g/L)	Liquid	[74]	
Rhodococcus sp. JG3	20	10	TSB+NaCl	Unclear	[75]	Data from digitized plot
Rhodococcus sp. JG3	5	40	TSB+NaCl	Unclear	[75]	data stated in paper (fig 1 legend)
Rhodococcus sp. JG3	0	60	TSB+NaCl	Unclear	[75]	data stated in paper (fig 1 legend)
Rhodococcus sp. JG3	-5	335	TSB+NaCl	Unclear	[75]	data stated in paper (fig 1 legend)
Rhodococcus sp. strain SGB1168-118	-10	8316		Liquid	[77]	Specific growth rate converted w factor of 0.693
Sporosarcina sp. B5	-5	1040	M9	Solid	[14]	Values provided in Table 1, converted from CTC-live cell counts
Sporosarcina sp. B5	-5	504	M9 + 5.7% NaCl	Liquid	[14]	Values provided in Table 1, converted from CTC-live cell counts
Sporosarcina sp. B5	-5	1040	M9 mineral media	Solid	[14]	Converted from values calculated in Table 1 w/ factor of 0.693;
Sporosarcina sp. B5	-5	504	M9 mineral media	Liquid	[14]	Converted from values calculated in Table 1 w/ factor of 0.693

* Specific growth rate converted via generation time (h) = 0.693/specific growth rate

† E. Firth, personal communication

‡ This paper
