# Supplementary material for: Metabolic Responses, Cell Recoverability, and Protein Signatures of Three Extremophiles: Sustained Life During Long-Term Subzero Incubations
Source: Microorganisms. 2025 Jan 24;13(2):251. doi: 10.3390/microorganisms13020251 (PMC11858272; doi:10.3390/microorganisms13020251)
Supplement: Supplementary file 1 [file microorganisms-13-00251-s001.zip › Table S3.pdf]

Table S3. Protein names and descriptions

Protein Name	Protein Description
tr|Q485N5|Q485N5_COLP3	Flagellin
tr|Q485N3|Q485N3_COLP3	Flagellin
sp|Q47UU9|EFTU_COLP3	Elongation factor Tu
tr|Q489V6|Q489V6_COLP3	Glutamine synthetase
tr|Q481C9|Q481C9_COLP3	Acetyl-CoA acetyltransferase
tr|Q47WT2|Q47WT2_COLP3	Putative granule-associated protein
tr|Q47WT3|Q47WT3_COLP3	Putative granule-associated protein
tr|Q47WS5|Q47WS5_COLP3	Acetoacetyl-CoA reductase
tr|Q482L4|Q482L4_COLP3	Phosphogluconate dehydratase
tr|Q482F8|Q482F8_COLP3	Glyceraldehyde-3-phosphate dehydrogenase
tr|Q488X3|Q488X3_COLP3	Nitrogen regulatory protein P-II
sp|P13717.2|NUCA_SERMA	(CONTAMINANT) Serratia marcescens benzonase nuclease
tr|Q484B2|Q484B2_COLP3	DNA-binding protein, HU family
tr|Q487N9|Q487N9_COLP3	TonB-dependent receptor
tr|Q483M6|Q483M6_COLP3	Porin
tr|Q47Y50|Q47Y50_COLP3	Transcriptional regulator, araC family
gi|136429|sp|P00761|TRYP_PIG	(CONTAMINANT) Trypsin precursor
tr|Q47UT1|Q47UT1_COLP3	Putative aconitate hydratase
tr|Q481D0|Q481D0_COLP3	Acetoacetyl-CoA reductase
tr|Q480B0|Q480B0_COLP3	Translation initiation factor IF-3
tr|Q487J5|Q487J5_COLP3	TonB-dependent receptor
tr|Q47W10|Q47W10_COLP3	Anti-sigma factor
tr|Q480K9|Q480K9_COLP3	DUF3015 domain-containing protein
tr|Q47WT1|Q47WT1_COLP3	Putative granule-associated protein
tr|Q480I5|Q480I5_COLP3	2-methylisocitrate lyase
tr|Q48AL6|Q48AL6_COLP3	Extracellular solute-binding protein, family 7
tr|Q485W8|Q485W8_COLP3	Extracellular solute-binding protein, family 7
tr|Q484A0|Q484A0_COLP3	Aldehyde dehydrogenase family protein
tr|Q487H2|Q487H2_COLP3	Oxaloacetate decarboxylase, alpha subunit
sp|Q486J6|GCSP1_COLP3	Glycine dehydrogenase (decarboxylating) 1
tr|Q488M7|Q488M7_COLP3	Cold-shock DNA-binding domain family protein
tr|Q484K6|Q484K6_COLP3	N-ethylmaleimide reductase
tr|Q48AA8|Q48AA8_COLP3	TonB-dependent receptor
tr|Q47X57|Q47X57_COLP3	Acetyl-coenzyme A synthetase
tr|Q47XW5|Q47XW5_COLP3	Electron transfer flavoprotein subunit beta
tr|Q480I6|Q480I6_COLP3	Citrate synthase
tr|Q47XP7|Q47XP7_COLP3	Uncharacterized protein
tr|Q47XP8|Q47XP8_COLP3	MotA/TolQ/ExbB proton channel family
tr|Q485C0|Q485C0_COLP3	Isovaleryl-CoA dehydrogenase, mitochondrial
tr|Q47X95|Q47X95_COLP3	Nitrogen regulatory protein P-II
tr|Q47XW4|Q47XW4_COLP3	Electron transfer flavoprotein, alpha subunit
tr|Q485L3|Q485L3_COLP3	Flagellar motor switch protein FliN
tr|Q47UD7|Q47UD7_COLP3	Nitrite reductase [NAD(P)H], large subunit
tr|Q489J9|Q489J9_COLP3	Glycosyl transferase

PRTC-peptide|YBR249C	ARO4 SGDID:S000000453, Chr II from 717994-716882, Genome Release 64-1-1, reverse complement, Verified ORF, "3-deoxy-D-arabino-heptulosonate-7-phosphate (DAHP) synthase, catalyzes the first step in aromatic amino acid biosynthesis and is feedback-inhibited by tyrosine or high concentrations of phenylalanine or tryptophan"
tr|Q480I9|Q480I9_COLP3	TonB-dependent receptor
tr|Q47XP6|Q47XP6_COLP3	TonB-dependent receptor
tr|Q481C1|Q481C1_COLP3	Extracellular ribonuclease/nuclease fusion protein
sp|Q487Z1|EFG1_COLP3	Elongation factor G 1
tr|Q47XP3|Q47XP3_COLP3	TonB-dependent receptor
tr|Q47UR0|Q47UR0_COLP3	Coproporphyrinogen-III oxidase
tr|Q485C5|Q485C5_COLP3	3-oxoadipate CoA-succinyl transferase, alpha subunit
sp|Q48AW0|ATPB_COLP3	ATP synthase subunit beta
sp|Q47WR3|TAL_COLP3	Transaldolase
tr|Q486W0|Q486W0_COLP3	TonB-dependent vitamin B12 receptor
sp|Q488Y0|ASTD_COLP3	N-succinylglutamate 5-semialdehyde dehydrogenase
tr|Q47VJ5|Q47VJ5_COLP3	Cold-shock DNA-binding domain family protein
tr|Q47VT0|Q47VT0_COLP3	Type IV pilin
tr|Q47UN8|Q47UN8_COLP3	Branched-chain-amino-acid aminotransferase
tr|Q47W28|Q47W28_COLP3	Z-ring associated protein G
tr|Q48AI8|Q48AI8_COLP3	Signal recognition particle receptor FtsY
tr|Q483V5|Q483V5_COLP3	Porin domain-containing protein
sp|Q482G2|IHFB_COLP3	Integration host factor subunit beta
sp|Q47VY2|GPMI_COLP3	2,3-bisphosphoglycerate-independent phosphoglycerate mutase
tr|Q481D4|Q481D4_COLP3	Cytochrome c family protein
tr|Q47YK6|Q47YK6_COLP3	Cytochrome c2
sp|Q489P3|ASSY_COLP3	Argininosuccinate synthase
tr|Q482L6|Q482L6_COLP3	Glucose-6-phosphate 1-dehydrogenase
tr|Q47YY1|Q47YY1_COLP3	Putative lipoprotein
tr|Q489M8|Q489M8_COLP3	Histidine kinase
tr|Q47UE3|Q47UE3_COLP3	diguanylate cyclase
tr|Q48A35|Q48A35_COLP3	PEP-CTERM protein-sorting domain-containing protein
tr|Q483R8|Q483R8_COLP3	Putative OmpA family protein
tr|Q482G3|Q482G3_COLP3	30S ribosomal protein S1
tr|Q485T4|Q485T4_COLP3	Peptidase S9 family protein
sp|Q485G9|EFTS_COLP3	Elongation factor Ts
tr|Q47X19|Q47X19_COLP3	TonB-dependent receptor
sp|Q47UW0|RPOC_COLP3	DNA-directed RNA polymerase subunit beta'
sp|Q488N6|GLYA1_COLP3	Serine hydroxymethyltransferase 1
tr|Q47WX2|Q47WX2_COLP3	Amidase, hydantoinase/carbamoylase family
sp|Q47Z78|PUR5_COLP3	Phosphoribosylformylglycinamidine cyclo-ligase
tr|Q48AJ4|Q48AJ4_COLP3	SapC family protein
tr|Q485C3|Q485C3_COLP3	Methylcrotonyl-CoA carboxylase, alpha subunit
sp|Q482T5|PNP_COLP3	Polyribonucleotide nucleotidyltransferase
tr|Q482K0|Q482K0_COLP3	3-oxoacyl-[acyl-carrier-protein] reductase
sp|Q488Y8|RPOA_COLP3	DNA-directed RNA polymerase subunit alpha
sp|Q47WR1|ENO_COLP3	Enolase

tr|Q47W81|Q47W81_COLP3	Bifunctional aspartokinase/homoserine dehydrogenase
tr|Q480M8|Q480M8_COLP3	Phospho-2-dehydro-3-deoxyheptonate aldolase
tr|Q482G7|Q482G7_COLP3	DNA gyrase subunit A
tr|Q48AH0|Q48AH0_COLP3	Thioredoxin
tr|Q47UC7|Q47UC7_COLP3	Peptidase S9 prolyl oligopeptidase catalytic domain-containing protein
tr|Q486J7|Q486J7_COLP3	Glycine cleavage system H protein
tr|Q485M7|Q485M7_COLP3	Flagellar secretion chaperone FliS
tr|Q485B8|Q485B8_COLP3	Acetyl-CoA acetyltransferase
tr|Q482S2|Q482S2_COLP3	Dihydrolipoyllysine-residue succinyltransferase component of 2-oxoglutarate dehydrogenase complex
tr|Q486S7|Q486S7_COLP3	Intimin/invasin family protein
sp|Q485G7|RRF_COLP3	Ribosome-recycling factor
tr|Q47Y86|Q47Y86_COLP3	Ribosome-binding ATPase YchF
tr|Q47W01|Q47W01_COLP3	Methyl-accepting chemotaxis protein
tr|Q489N5|Q489N5_COLP3	Type IV pilus biogenesis protein PilQ
tr|Q485H9|Q485H9_COLP3	D-3-phosphoglycerate dehydrogenase
sp|Q47VJ4|MURA_COLP3	UDP-N-acetylglucosamine 1-carboxyvinyltransferase
tr|Q47VC8|Q47VC8_COLP3	Transcriptional regulatory protein OmpR
tr|Q47WH2|Q47WH2_COLP3	GDP-mannose 4,6-dehydratase
tr|Q47UB4|Q47UB4_COLP3	Aminotransferase
sp|Q47XG4|GLYA3_COLP3	Serine hydroxymethyltransferase 3
tr|Q47W64|Q47W64_COLP3	Oxidoreductase, short chain dehydrogenase/reductase family
tr|Q482L3|Q482L3_COLP3	2-dehydro-3-deoxy-phosphogluconate aldolase
tr|Q47XR6|Q47XR6_COLP3	TonB-dependent receptor
tr|Q47YM1|Q47YM1_COLP3	3-hydroxyisobutyrate dehydrogenase
tr|Q487J2|Q487J2_COLP3	SapC family protein
tr|Q480M6|Q480M6_COLP3	DUF3083 family protein
tr|Q47Z12|Q47Z12_COLP3	DUF3466 family protein
sp|Q47V01|SYY_COLP3	Tyrosine--tRNA ligase
sp|Q47WQ9|PYRG_COLP3	CTP synthase
tr|Q485T2|Q485T2_COLP3	Neutral zinc metallopeptidase, M13 family
sp|Q47U38|MNMG_COLP3	tRNA uridine 5-carboxymethylaminomethyl modification enzyme MnmG
tr|Q489U7|Q489U7_COLP3	CheC-like family protein
tr|Q48AP7|Q48AP7_COLP3	methylmalonate-semialdehyde dehydrogenase (CoA acylating)
tr|Q482L7|Q482L7_COLP3	Transcriptional regulator, RpiR family
tr|Q48AG3|Q48AG3_COLP3	Coenzyme A biosynthesis bifunctional protein CoaBC
tr|Q47X43|Q47X43_COLP3	Alpha-2-macroglobulin
tr|Q47XV4|Q47XV4_COLP3	TonB-dependent receptor
tr|Q489V9|Q489V9_COLP3	DNA-binding transcriptional regulator NtrC
tr|Q484J6|Q484J6_COLP3	MaoC domain protein
tr|Q47WK6|Q47WK6_COLP3	Response regulator/phosphatase
tr|Q481D9|Q481D9_COLP3	ATP-binding protein
tr|Q47W12|Q47W12_COLP3	Replicative DNA helicase
tr|Q47W29|Q47W29_COLP3	Cell division protein ZapE
tr|Q480H8|Q480H8_COLP3	Putative gluconeogenesis factor
sp|Q47U36|MNME_COLP3	tRNA modification GTPase MnmE

tr|Q47VX1|Q47VX1_COLP3	DUF3581 domain-containing protein
tr|Q47YX8|Q47YX8_COLP3	Putative nitrate ABC transporter, periplasmic nitrate-binding protein
tr|Q489J7|Q489J7_COLP3	Kinase
tr|Q489P6|Q489P6_COLP3	N-acetyl-gamma-glutamyl-phosphate reductase
tr|Q485P4|Q485P4_COLP3	Basal-body rod modification protein FlgD
tr|Q487L6|Q487L6_COLP3	Magnesium transporter MgtE intracellular domain-containing protein
tr|Q47YY0|Q47YY0_COLP3	Putative nitrate ABC transporter, ATP-binding protein
tr|Q488P2|Q488P2_COLP3	prolyl aminopeptidase
tr|Q483G2|Q483G2_COLP3	Amidohydrolase 3 domain-containing protein
tr|Q485Z0|Q485Z0_COLP3	Oxidoreductase, short-chain dehydrogenase/reductase family
sp|Q480C7|UVRB_COLP3	UvrABC system protein B
tr|Q481X2|Q481X2_COLP3	VCBS repeat protein
tr|Q487B5|Q487B5_COLP3	PilZ domain-containing protein
tr|Q484L5|Q484L5_COLP3	Transcriptional regulator, LysR family
sp|Q482K2|PLSX_COLP3	Phosphate acyltransferase
tr|Q488V9|Q488V9_COLP3	Aminoglycoside phosphotransferase domain-containing protein
tr|Q487S7|Q487S7_COLP3	histidine kinase
tr|Q483G1|Q483G1_COLP3	Pyruvate ferredoxin/flavodoxin oxidoreductase
tr|Q485Y0|Q485Y0_COLP3	Transcriptional regulator, LysR family
tr|Q47Y42|Q47Y42_COLP3	Transcriptional regulator, LysR family
tr|Q47WV3|Q47WV3_COLP3	Uncharacterized protein
tr|Q47UE1|Q47UE1_COLP3	Transcriptional regulator, DeoR family
tr|Q47V24|Q47V24_COLP3	Sigma-54 dependent DNA-binding response regulator, Fis family
tr|Q482R9|Q482R9_COLP3	FRG domain-containing protein
tr|Q489D3|Q489D3_COLP3	Uncharacterized protein
tr|Q489H0|Q489H0_COLP3	RNA pseudouridine synthase family protein
tr|Q47XV7|Q47XV7_COLP3	Gluconokinase
tr|Q47Z23|Q47Z23_COLP3	Monooxygenase family protein
tr|Q47YR5|Q47YR5_COLP3	von Willebrand factor type A domain protein
tr|Q47Z33|Q47Z33_COLP3	Glycosyl transferase, WecB/TagA/CpsF family
tr|Q47ZB5|Q47ZB5_COLP3	Uncharacterized protein
tr|Q485D4|Q485D4_COLP3	Uncharacterized protein
tr|Q48AC8|Q48AC8_COLP3	Competence protein, homolog
gi|27769210|gb|AAH42174.1|	(CONTAMINANT) Keratin 4 [Homo sapiens]
PRTC-peptide|YDR226W	ADK1 SGDID:S000002634, Chr IV from 916486-917154, Genome Release 64-1-1, Verified ORF, "Adenylate kinase, required for purine metabolism; localized to the cytoplasm and the mitochondria; lacks cleavable signal sequence"
gi|2136983|pir||I46732	(CONTAMINANT) Ig gamma heavy chain constant region - rabbit (fragment)
tr|Q47X18|Q47X18_COLP3	Putative lipoprotein
tr|Q47WF2|Q47WF2_COLP3	Nitrite reductase
sp|Q485H0|RS2_COLP3	Small ribosomal subunit protein uS2
sp|Q488M6|SYV_COLP3	Valine--tRNA ligase
sp|Q487Z9|RS3_COLP3	Small ribosomal subunit protein uS3

sp|Q47WV1|RL19_COLP3	Large ribosomal subunit protein bL19
sp|Q48AW2|ATPA_COLP3	ATP synthase subunit alpha
sp|Q47UV9|RPOB_COLP3	DNA-directed RNA polymerase subunit beta
tr|Q47WI0|Q47WI0_COLP3	PspA/IM30 family protein
sp|Q48A14|STHA_COLP3	Soluble pyridine nucleotide transhydrogenase
sp|Q47XR0|Y3743_COLP3	Nucleoid-associated protein CPS_3743
sp|Q484Q5|SYL_COLP3	Leucine--tRNA ligase
tr|Q482H8|Q482H8_COLP3	Denitrification regulatory protein nirQ
tr|Q485C1|Q485C1_COLP3	Methylcrotonyl CoA carboxylase, beta subunit
tr|Q47YI4|Q47YI4_COLP3	Peptidyl-prolyl cis-trans isomerase
tr|Q47YM2|Q47YM2_COLP3	Methylmalonate-semialdehyde dehydrogenase
sp|Q483C7|SYDND_COLP3	Aspartate--tRNA(Asp/Asn) ligase
tr|Q47UZ6|Q47UZ6_COLP3	Nitrous-oxide reductase
tr|Q488X8|Q488X8_COLP3	Acetylornithine aminotransferase
tr|Q482K8|Q482K8_COLP3	Ribonuclease E
tr|Q489A8|Q489A8_COLP3	Large ribosomal subunit assembly factor BipA
sp|Q487G4|NAPA_COLP3	Periplasmic nitrate reductase
tr|Q486R4|Q486R4_COLP3	Phosphate regulon transcriptional regulatory protein PhoB
tr|Q480I7|Q480I7_COLP3	Aconitate hydratase
tr|Q47VT4|Q47VT4_COLP3	Ubiquinol-cytochrome c reductase iron-sulfur subunit
tr|Q480K2|Q480K2_COLP3	Outer membrane lipoprotein-sorting protein
tr|Q487C0|Q487C0_COLP3	Methionine synthase
sp|Q47V96|GSA_COLP3	Glutamate-1-semialdehyde 2,1-aminomutase
tr|Q48AH1|Q48AH1_COLP3	ATP-dependent RNA helicase RhlB
tr|Q489B8|Q489B8_COLP3	Putative lipoprotein
tr|Q485I7|Q485I7_COLP3	Efflux transporter, MFP subunit, AcrA/E family
tr|Q47X99|Q47X99_COLP3	Chaperone protein ClpB
tr|Q47YB9|Q47YB9_COLP3	Tryptophan synthase alpha chain
tr|Q480B4|Q480B4_COLP3	Glu/Leu/Phe/Val dehydrogenase
tr|Q486C4|Q486C4_COLP3	Putative Xaa-Pro dipeptidase
tr|Q485P3|Q485P3_COLP3	Flagellar hook protein FlgE
tr|Q487E1|Q487E1_COLP3	Na(+)-translocating NADH-quinone reductase subunit A
sp|Q47VY6|GPDA_COLP3	Glycerol-3-phosphate dehydrogenase [NAD(P)+]
sp|Q47Z40|SYQ_COLP3	Glutamine--tRNA ligase
tr|Q47XB9|Q47XB9_COLP3	ATP phosphoribosyltransferase
sp|Q47UW3|EFG2_COLP3	Elongation factor G 2
sp|Q47XI6|DNAK2_COLP3	Chaperone protein DnaK 2
tr|Q482U0|Q482U0_COLP3	Transcription termination/antitermination protein NusA
sp|Q47XA8|KAD_COLP3	Adenylate kinase
sp|Q482S1|SUCC_COLP3	Succinate--CoA ligase [ADP-forming] subunit beta
tr|Q47Z76|Q47Z76_COLP3	DUF3108 domain-containing protein
tr|Q486P0|Q486P0_COLP3	Isocitrate lyase
tr|Q482S3|Q482S3_COLP3	oxoglutarate dehydrogenase (succinyl-transferring)
tr|Q482S0|Q482S0_COLP3	Succinate--CoA ligase [ADP-forming] subunit alpha
tr|Q47Z72|Q47Z72_COLP3	Putative lipoprotein
tr|Q47VP0|Q47VP0_COLP3	Efflux transporter, RND family, MFP subunit subfamily
tr|Q482P1|Q482P1_COLP3	Cysteine synthase
tr|Q485X5|Q485X5_COLP3	Oxidoreductase, FAD/FMN-binding
sp|Q487H5|SYA_COLP3	Alanine--tRNA ligase

tr|Q485M0|Q485M0_COLP3	Flagellar motor switch protein FliG
sp|Q47XA7|HTPG_COLP3	Chaperone protein HtpG
sp|Q47UV6|RL1_COLP3	Large ribosomal subunit protein uL1
tr|Q486B8|Q486B8_COLP3	Isocitrate dehydrogenase [NADP]
tr|Q480P6|Q480P6_COLP3	Alanine dehydrogenase
tr|Q47YJ4|Q47YJ4_COLP3	ATP-dependent zinc metalloprotease FtsH
sp|Q47XK5|ACCD_COLP3	Acetyl-coenzyme A carboxylase carboxyl transferase subunit beta
tr|Q47UN6|Q47UN6_COLP3	L-threonine dehydratase
tr|Q47WY0|Q47WY0_COLP3	Sarcosine oxidase, delta subunit
tr|Q47US7|Q47US7_COLP3	Acetyltransferase component of pyruvate dehydrogenase complex
sp|Q47VT2|RS9_COLP3	Small ribosomal subunit protein uS9
tr|Q47YC1|Q47YC1_COLP3	Multifunctional fusion protein
tr|Q47VM3|Q47VM3_COLP3	Glutamate synthase, large subunit
tr|Q47VP8|Q47VP8_COLP3	Acyl-CoA dehydrogenase family protein
tr|Q47Y07|Q47Y07_COLP3	Polysaccharide biosynthesis/export protein
tr|Q47UA6|Q47UA6_COLP3	DNA polymerase I
tr|Q485E1|Q485E1_COLP3	2-oxoisovalerate dehydrogenase subunit alpha
sp|Q486R1|ISPH_COLP3	4-hydroxy-3-methylbut-2-enyl diphosphate reductase
tr|Q48AH6|Q48AH6_COLP3	Delta-aminolevulinic acid dehydratase
tr|Q47Y13|Q47Y13_COLP3	Phosphate-specific transport system accessory protein PhoU
sp|Q485F2|ACCA_COLP3	Acetyl-coenzyme A carboxylase carboxyl transferase subunit alpha
tr|Q47VR5|Q47VR5_COLP3	Cell division protein FtsZ
tr|Q485K1|Q485K1_COLP3	histidine kinase
tr|Q48A19|Q48A19_COLP3	Protein HflC
tr|Q488E7|Q488E7_COLP3	S-formylglutathione hydrolase
sp|Q47XN8|PHS_COLP3	Putative pterin-4-alpha-carbinolamine dehydratase
tr|Q486U5|Q486U5_COLP3	Acyl-CoA dehydrogenase family protein
tr|Q47ZI1|Q47ZI1_COLP3	Putative peptidase, M28 family
tr|Q47YC4|Q47YC4_COLP3	Anthranilate synthase component 1
tr|Q47X11|Q47X11_COLP3	WD40-like beta Propeller containing protein
tr|Q484N1|Q484N1_COLP3	Xanthine/uracil permease family protein
tr|Q488K4|Q488K4_COLP3	Putative glutamate synthase, ferredoxin-dependent
tr|Q47YP4|Q47YP4_COLP3	ATP-dependent RNA helicase, DEAD box family
tr|Q486B3|Q486B3_COLP3	Adenine deaminase
tr|Q482V7|Q482V7_COLP3	Putative lipoprotein
tr|Q485C2|Q485C2_COLP3	Enoyl-CoA hydratase/isomerase family protein
tr|Q483E8|Q483E8_COLP3	Polysaccharide biosynthesis protein
sp|Q48AS7|DNAA_COLP3	Chromosomal replication initiator protein DnaA
sp|Q47ZS4|SYFA_COLP3	Phenylalanine--tRNA ligase alpha subunit
sp|Q485K0|CHEB_COLP3	Protein-glutamate methylesterase/protein-glutamine glutaminase
tr|Q47UT3|Q47UT3_COLP3	Aconitate hydratase B
tr|Q7WVY1|Q7WVY1_COLP3	Aminopeptidase N (Fragment)
tr|Q487T0|Q487T0_COLP3	TonB-dependent receptor
tr|Q483U6|Q483U6_COLP3	Response regulator
tr|Q47WU4|Q47WU4_COLP3	Signal recognition particle protein

sp|Q482T7|TRUB_COLP3	tRNA pseudouridine synthase B
tr|Q48AJ3|Q48AJ3_COLP3	Uncharacterized protein
tr|Q47WX0|Q47WX0_COLP3	NADPH-dependent F420 reductase
sp|Q47UE0|GLMU_COLP3	Bifunctional protein GlmU
tr|Q47WM5|Q47WM5_COLP3	Trypsin family protein
tr|Q47WR7|Q47WR7_COLP3	TIGR03545 family protein
tr|Q47U98|Q47U98_COLP3	oligopeptidase A
tr|Q488X9|Q488X9_COLP3	Arginine N-succinyltransferase
tr|Q489D4|Q489D4_COLP3	Tyrosine-protein kinase
tr|Q47VZ3|Q47VZ3_COLP3	Small ribosomal subunit biogenesis GTPase RsgA
sp|Q47XL9|CLPX_COLP3	ATP-dependent Clp protease ATP-binding subunit ClpX
tr|Q47VL1|Q47VL1_COLP3	Octaprenyl-diphosphate synthase
tr|Q48AG0|Q48AG0_COLP3	Putative site-specific recombinase, phage integrase family
tr|Q48AD9|Q48AD9_COLP3	ParA family protein
sp|Q47VL2|RL21_COLP3	Large ribosomal subunit protein bL21
tr|Q47WK7|Q47WK7_COLP3	Secretion protein, HlyD family
tr|Q47Z43|Q47Z43_COLP3	Acetolactate synthase small subunit
tr|Q47VC3|Q47VC3_COLP3	Porin domain-containing protein
tr|Q486Z2|Q486Z2_COLP3	serine O-acetyltransferase
tr|Q47Y23|Q47Y23_COLP3	Amidohydrolase family protein
tr|Q47WG4|Q47WG4_COLP3	2-isopropylmalate synthase
tr|Q48A78|Q48A78_COLP3	Putative Xaa-Pro dipeptidase
tr|Q483M2|Q483M2_COLP3	Exodeoxyribonuclease I
tr|Q48AB5|Q48AB5_COLP3	Thioredoxin domain-containing protein
tr|Q486A6|Q486A6_COLP3	ABC transporter, ATP-binding protein
tr|Q47YD5|Q47YD5_COLP3	Prolyl oligopeptidase family protein
tr|Q47Z48|Q47Z48_COLP3	alanine transaminase
tr|Q47ZS2|Q47ZS2_COLP3	FAD binding protein
tr|Q47VW8|Q47VW8_COLP3	S-adenosylmethionine-dependent methyltransferase domain-containing protein
tr|Q485X4|Q485X4_COLP3	Uncharacterized protein
tr|Q487J3|Q487J3_COLP3	Putative tryptophan halogenase
tr|Q483Z8|Q483Z8_COLP3	Quinoprotein alcohol dehydrogenase
tr|Q48AX1|Q48AX1_COLP3	HemY protein
sp|Q47VQ1|RSMH_COLP3	Ribosomal RNA small subunit methyltransferase H
tr|Q482Z0|Q482Z0_COLP3	Thrombospondin type 3 repeat family protein/Calx-beta domain protein
tr|Q489S4|Q489S4_COLP3	Sel1 domain protein repeat-containing protein
sp|Q48AM4|TDH_COLP3	L-threonine 3-dehydrogenase
tr|Q47U50|Q47U50_COLP3	Outer membrane protein beta-barrel domain-containing protein
tr|Q47YP1|Q47YP1_COLP3	Snf2 family protein
tr|Q480Z7|Q480Z7_COLP3	RNA helicase
tr|Q47VB7|Q47VB7_COLP3	Putative membrane protein
tr|Q486P9|Q486P9_COLP3	Acyl-homoserine lactone acylase QuiP
sp|Q481U4|Y2458_COLP3	UPF0145 protein CPS_2458
tr|Q48AR6|Q48AR6_COLP3	HAD-superfamily hydrolase, subfamily IA, variant 1 family protein
tr|Q47WA0|Q47WA0_COLP3	Sigma-54 dependent response regulator
tr|Q482N4|Q482N4_COLP3	Acyl-CoA dehydrogenase family protein

tr|Q47YL8|Q47YL8_COLP3	Long-chain-fatty-acid--CoA ligase
tr|Q481X3|Q481X3_COLP3	Membrane fusion protein (MFP) family protein
tr|Q47XT1|Q47XT1_COLP3	Cellobiose phosphorylase
tr|Q47UY1|Q47UY1_COLP3	Nitroreductase family protein
tr|Q47WI8|Q47WI8_COLP3	Lipid A biosynthesis acyltransferase
tr|Q481R3|Q481R3_COLP3	Cupin type-2 domain-containing protein
tr|Q47U40|Q47U40_COLP3	ParA family protein
tr|Q483M3|Q483M3_COLP3	C4-dicarboxylate transport transcriptional regulatory protein
tr|Q47VF1|Q47VF1_COLP3	MSHA biogenesis protein MshE
tr|Q487R5|Q487R5_COLP3	Phosphate-selective porin O and P
tr|Q47VW6|Q47VW6_COLP3	HDOD domain-containing protein
tr|Q48AQ8|Q48AQ8_COLP3	Fatty acid cis/trans isomerase
tr|Q47YW2|Q47YW2_COLP3	Uncharacterized protein
tr|Q489C7|Q489C7_COLP3	asparagine synthase (glutamine-hydrolyzing)
tr|Q47VK2|Q47VK2_COLP3	Co-chaperone protein DjlA
tr|Q486F1|Q486F1_COLP3	Aldehyde dehydrogenase (NAD) family protein
tr|Q47YM8|Q47YM8_COLP3	Putative branched-chain amino acid ABC transporter, ATP-binding protein
sp|Q48AS9|FMT_COLP3	Methionyl-tRNA formyltransferase
tr|Q47WZ7|Q47WZ7_COLP3	Iron-sulfur cluster-binding protein
tr|Q485Y1|Q485Y1_COLP3	Zinc-type alcohol dehydrogenase-like protein
tr|Q48AR2|Q48AR2_COLP3	DNA helicase
tr|Q486R3|Q486R3_COLP3	histidine kinase
tr|Q485J4|Q485J4_COLP3	DUF2802 domain-containing protein
tr|Q484B3|Q484B3_COLP3	Mur ligase family protein
tr|Q484D7|Q484D7_COLP3	AcrB/AcrD/AcrF family protein
tr|Q47Y19|Q47Y19_COLP3	Protein-methionine-sulfoxide reductase catalytic subunit MsrP
tr|Q47VC9|Q47VC9_COLP3	histidine kinase
tr|Q47Y03|Q47Y03_COLP3	tRNA pseudouridine synthase C
tr|Q47U53|Q47U53_COLP3	histidine kinase
tr|Q480W1|Q480W1_COLP3	NAD-glutamate dehydrogenase family protein
tr|Q486S9|Q486S9_COLP3	SohB protein, peptidase U7 family
tr|Q47WQ3|Q47WQ3_COLP3	MazG family protein
tr|Q47Y63|Q47Y63_COLP3	RmuC-domain protein
tr|Q47U93|Q47U93_COLP3	Uncharacterized protein
tr|Q47UD9|Q47UD9_COLP3	Nitrate reductase
tr|Q47UG5|Q47UG5_COLP3	Transcriptional regulator, AsnC family
tr|Q47WB8|Q47WB8_COLP3	LysM domain protein
tr|Q47WF7|Q47WF7_COLP3	Multifunctional CCA protein
tr|Q481A1|Q481A1_COLP3	PKD domain protein
tr|Q482I2|Q482I2_COLP3	Lysozyme inhibitor LprI N-terminal domain-containing protein
tr|Q488T4|Q488T4_COLP3	histidine kinase
tr|Q482I7|Q482I7_COLP3	ABC transporter, permease protein
tr|Q484I6|Q484I6_COLP3	ATP-dependent peptidase, M41 family
tr|Q47VB2|Q47VB2_COLP3	Membrane protein
tr|Q47UA8|Q47UA8_COLP3	ATP-dependent DNA helicase RecG
tr|Q47Y46|Q47Y46_COLP3	DUF805 domain-containing protein

tr|Q48AP6|Q48AP6_COLP3	Omega-amino acid--pyruvate aminotransferase
tr|Q483U7|Q483U7_COLP3	histidine kinase
tr|Q486J1|Q486J1_COLP3	Metallo-beta-lactamase family protein
tr|Q481F5|Q481F5_COLP3	Cell division protein FtsZ
tr|Q47ZY4|Q47ZY4_COLP3	tRNA-guanine(15) transglycosylase-like domain-containing protein
tr|Q487M1|Q487M1_COLP3	Transcriptional regulator, LysR family
tr|Q47ZI7|Q47ZI7_COLP3	Amidohydrolase family protein
tr|Q47V40|Q47V40_COLP3	Iron-sulfur cluster-binding protein
tr|Q47Z08|Q47Z08_COLP3	DNA-binding protein
tr|Q481M3|Q481M3_COLP3	Transcriptional regulator, LysR family
tr|Q484F3|Q484F3_COLP3	Cytochrome c-type protein
tr|Q488N4|Q488N4_COLP3	Riboflavin biosynthesis protein RibD
tr|Q480A6|Q480A6_COLP3	Ferredoxin
tr|Q47Y38|Q47Y38_COLP3	aminodeoxychorismate synthase
tr|Q47ZM5|Q47ZM5_COLP3	Porin domain-containing protein
tr|Q48AT7|Q48AT7_COLP3	Putative soluble lytic murein transglycosylase
sp|Q487E8|ISPF_COLP3	2-C-methyl-D-erythritol 2,4-cyclodiphosphate synthase
tr|Q480T5|Q480T5_COLP3	Putative alpha-methylacyl-CoA racemase
tr|Q486F7|Q486F7_COLP3	Cyclic nucleotide-binding domain-containing protein
tr|Q47W32|Q47W32_COLP3	2-amino-4-hydroxy-6-hydroxymethyldihydropteridine pyrophosphokinase
tr|Q47W45|Q47W45_COLP3	TPR domain protein
tr|Q480E9|Q480E9_COLP3	Rad50/SbcC-type AAA domain-containing protein
tr|Q486C0|Q486C0_COLP3	HDOD domain-containing protein
tr|Q486N4|Q486N4_COLP3	Rhodanese domain-containing protein
tr|Q482C5|Q482C5_COLP3	Sulfatase family protein
tr|Q482B7|Q482B7_COLP3	Putative surface protein
tr|Q487C2|Q487C2_COLP3	MltA-interacting MipA family protein
tr|Q47YL2|Q47YL2_COLP3	Outer membrane lipoprotein-sorting protein
tr|Q487I8|Q487I8_COLP3	Glucose/galactose transporter family protein
tr|Q47YX7|Q47YX7_COLP3	Putative response regulator NasT
tr|Q47VY1|Q47VY1_COLP3	Peptidase, M23/37 family
tr|Q488U4|Q488U4_COLP3	Oxidoreductase, GMC family
tr|Q47YM6|Q47YM6_COLP3	histidine kinase
tr|Q47X73|Q47X73_COLP3	Pyridoxamine 5'-phosphate oxidase / oxidoreductase, NAD-dependent
tr|Q47YT7|Q47YT7_COLP3	Putative permease
tr|Q47W21|Q47W21_COLP3	Amidohydrolase family protein
tr|Q47YZ7|Q47YZ7_COLP3	VWA containing CoxE family protein
tr|Q483C0|Q483C0_COLP3	Sigma-54 dependent DNA-binding response regulator, Fis family
tr|Q48AM2|Q48AM2_COLP3	Putative glpG protein
tr|Q482A9|Q482A9_COLP3	DUF1800 domain-containing protein
tr|Q47VG9|Q47VG9_COLP3	Putative protease
tr|Q489B7|Q489B7_COLP3	Putative lipoprotein
tr|Q483P0|Q483P0_COLP3	Transcriptional regulator, Crp/Fnr family
tr|Q488W4|Q488W4_COLP3	histidine kinase
tr|Q487E3|Q487E3_COLP3	Putative lipoprotein NlpD
tr|Q47ZZ0|Q47ZZ0_COLP3	Acetyltransferase, GNAT family

tr|Q487M6|Q487M6_COLP3	Transcriptional regulator, LysR family
tr|Q481B5|Q481B5_COLP3	ABC transporter, ATP-binding protein
tr|Q47YW8|Q47YW8_COLP3	Putative lipoprotein
tr|Q485K6|Q485K6_COLP3	Flagellar biosynthesis protein FlhF
tr|Q47UF9|Q47UF9_COLP3	histidine kinase
tr|Q484S5|Q484S5_COLP3	DUF3422 domain-containing protein
tr|Q47XU6|Q47XU6_COLP3	Sugar:cation symporter family protein
tr|Q485S8|Q485S8_COLP3	PpiC domain-containing protein
tr|Q47X35|Q47X35_COLP3	Transcriptional regulator, LysR family
tr|Q488S8|Q488S8_COLP3	diguanylate cyclase
tr|Q487M0|Q487M0_COLP3	pullulanase
tr|Q47ZI6|Q47ZI6_COLP3	Transcriptional regulator, LysR family
tr|Q48A89|Q48A89_COLP3	BPL/LPL catalytic domain-containing protein
tr|Q47X21|Q47X21_COLP3	Transcriptional regulator, AraC family
tr|Q488P1|Q488P1_COLP3	ATP-binding transport protein NatA
tr|Q47XU5|Q47XU5_COLP3	Sugar:cation symporter family protein
tr|Q483C1|Q483C1_COLP3	Putative ABC transporter, permease protein
tr|Q480F4|Q480F4_COLP3	Peptidase
tr|Q48A80|Q48A80_COLP3	Phosphate-selective porin O and P
tr|Q47W95|Q47W95_COLP3	Sel1 domain protein repeat-containing protein
tr|Q47YV0|Q47YV0_COLP3	TonB-dependent receptor
tr|Q47UG3|Q47UG3_COLP3	Substrate-binding transcriptional regulator, LysR family
tr|Q47VV8|Q47VV8_COLP3	Efflux transporter, RND family, MFP subunit
tr|Q47V23|Q47V23_COLP3	histidine kinase
tr|Q47ZG2|Q47ZG2_COLP3	Oxidoreductase, FAD/FMN-binding
tr|Q480Z5|Q480Z5_COLP3	Methyl-accepting chemotaxis protein
tr|Q47XD8|Q47XD8_COLP3	Oxidoreductase, alpha/molybdopterin subunit
tr|Q47VQ8|Q47VQ8_COLP3	Probable peptidoglycan glycosyltransferase FtsW
tr|Q486I8|Q486I8_COLP3	Metallo-beta-lactamase family protein
tr|Q483S4|Q483S4_COLP3	Efflux transporter, RND family, MFP subunit
tr|Q485N8|Q485N8_COLP3	Peptidoglycan hydrolase FlgJ
tr|Q47ZU9|Q47ZU9_COLP3	CBS domain protein
tr|Q47YL6|Q47YL6_COLP3	Putative membrane protein
tr|Q484B8|Q484B8_COLP3	NRDE family protein
tr|Q480D8|Q480D8_COLP3	ATP-grasp domain-containing protein
tr|Q47ZL2|Q47ZL2_COLP3	Uncharacterized protein
tr|Q487B3|Q487B3_COLP3	Virulence factor, hemolysin regulator
tr|Q482D8|Q482D8_COLP3	Glycosyl hydrolase, family 5
tr|Q481L7|Q481L7_COLP3	Putative membrane protein
tr|Q483K6|Q483K6_COLP3	histidine kinase
tr|Q488E6|Q488E6_COLP3	diguanylate cyclase
tr|Q488J2|Q488J2_COLP3	Transcription factor zinc-finger domain-containing protein
tr|Q47WE2|Q47WE2_COLP3	Uncharacterized protein
tr|Q48AC7|Q48AC7_COLP3	Peptidase M14 carboxypeptidase A domain-containing protein
tr|Q47US1|Q47US1_COLP3	TIGR02281 family clan AA aspartic protease
tr|Q480D9|Q480D9_COLP3	Orn/DAP/Arg decarboxylase, family 2
tr|Q483I6|Q483I6_COLP3	Aldehyde dehydrogenase family protein
tr|Q484W9|Q484W9_COLP3	Putative tRNA-(MS[2]IO[6]A)-hydroxylase
tr|Q47ZT7|Q47ZT7_COLP3	Transcriptional regulator, AraC family

tr|Q480G5|Q480G5_COLP3	Nuclease SbcCD subunit D
tr|Q486Y3|Q486Y3_COLP3	GGDEF/GAF/EAL domain protein
tr|Q47YR1|Q47YR1_COLP3	Transcriptional regulator, TetR family
tr|Q482C3|Q482C3_COLP3	Glycosyl hydrolase, family 10
tr|Q48AU6|Q48AU6_COLP3	Putative DNA processing protein DprA
tr|Q47VI9|Q47VI9_COLP3	Intermembrane phospholipid transport system permease protein MlaE
tr|Q484J0|Q484J0_COLP3	Protein kinase domain protein
tr|Q485R6|Q485R6_COLP3	Transcriptional regulator, LysR family
tr|Q48AN1|Q48AN1_COLP3	Serine aminopeptidase S33 domain-containing protein
tr|Q47VD8|Q47VD8_COLP3	General secretion pathway protein H
tr|Q47ZU0|Q47ZU0_COLP3	DUF1415 domain-containing protein
tr|Q484V0|Q484V0_COLP3	Metallo-beta-lactamase family protein
tr|Q47WA9|Q47WA9_COLP3	FeS assembly protein IscX
tr|Q489K8|Q489K8_COLP3	MmcQ-like protein
tr|Q47WY8|Q47WY8_COLP3	Sulfur carrier protein FdhD
tr|Q486E5|Q486E5_COLP3	Uncharacterized protein
tr|Q482N8|Q482N8_COLP3	Putative membrane protein
tr|Q47V19|Q47V19_COLP3	FAD-binding oxidoreductase
tr|Q485U4|Q485U4_COLP3	Uncharacterized protein
tr|Q483X9|Q483X9_COLP3	Transcriptional regulator, LysR family
tr|Q47UY9|Q47UY9_COLP3	Regulator of ribonuclease activity B domain-containing protein
tr|Q481M0|Q481M0_COLP3	Uncharacterized protein
tr|Q47YL0|Q47YL0_COLP3	DUF1145 domain-containing protein
gi|21961605|gb|AAH34697.1|	(CONTAMINANT) Keratin 10 [Homo sapiens]
gi|34783124|gb|AAH02539.2|	(CONTAMINANT) Keratin 19 [Homo sapiens]
gi|1346343|sp|P04264|K2C1_HUMAN	(CONTAMINANT) Keratin, type II cytoskeletal 1 (Cytokeratin 1) (K1) (CK 1) (67 kDa cytokeratin) (Hair alpha protein)
gi|9790345|ref|NP_062908.1|	(CONTAMINANT) polyprotein [Tobacco etch virus]
gi|15080273|gb|AAH11901.1|, gi|33991652|gb|AAH56421.1|	(CONTAMINANT) Keratin 17 [Homo sapiens]
gi|39794653|gb|AAH63697.1|	(CONTAMINANT) Keratin 1 [Homo sapiens]
