# Supplementary figures and images for: Metabolic Responses, Cell Recoverability, and Protein Signatures of Three Extremophiles: Sustained Life During Long-Term Subzero Incubations
Source: Microorganisms. 2025 Jan 24;13(2):251. doi: 10.3390/microorganisms13020251 (PMC11858272; doi:10.3390/microorganisms13020251)

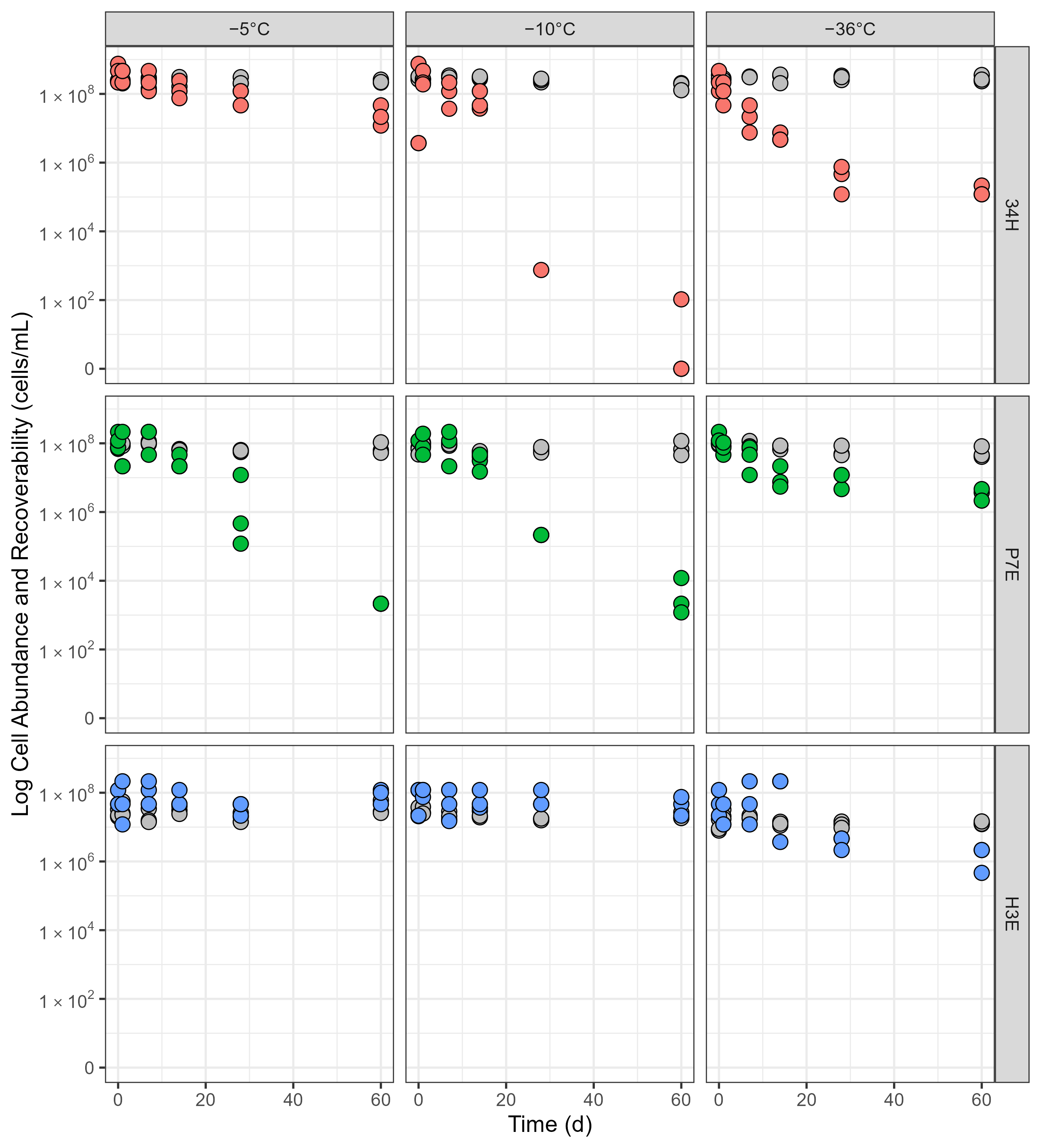

Supplement: Supplementary file 1 [file microorganisms-13-00251-s001.zip › Figure S1.png]

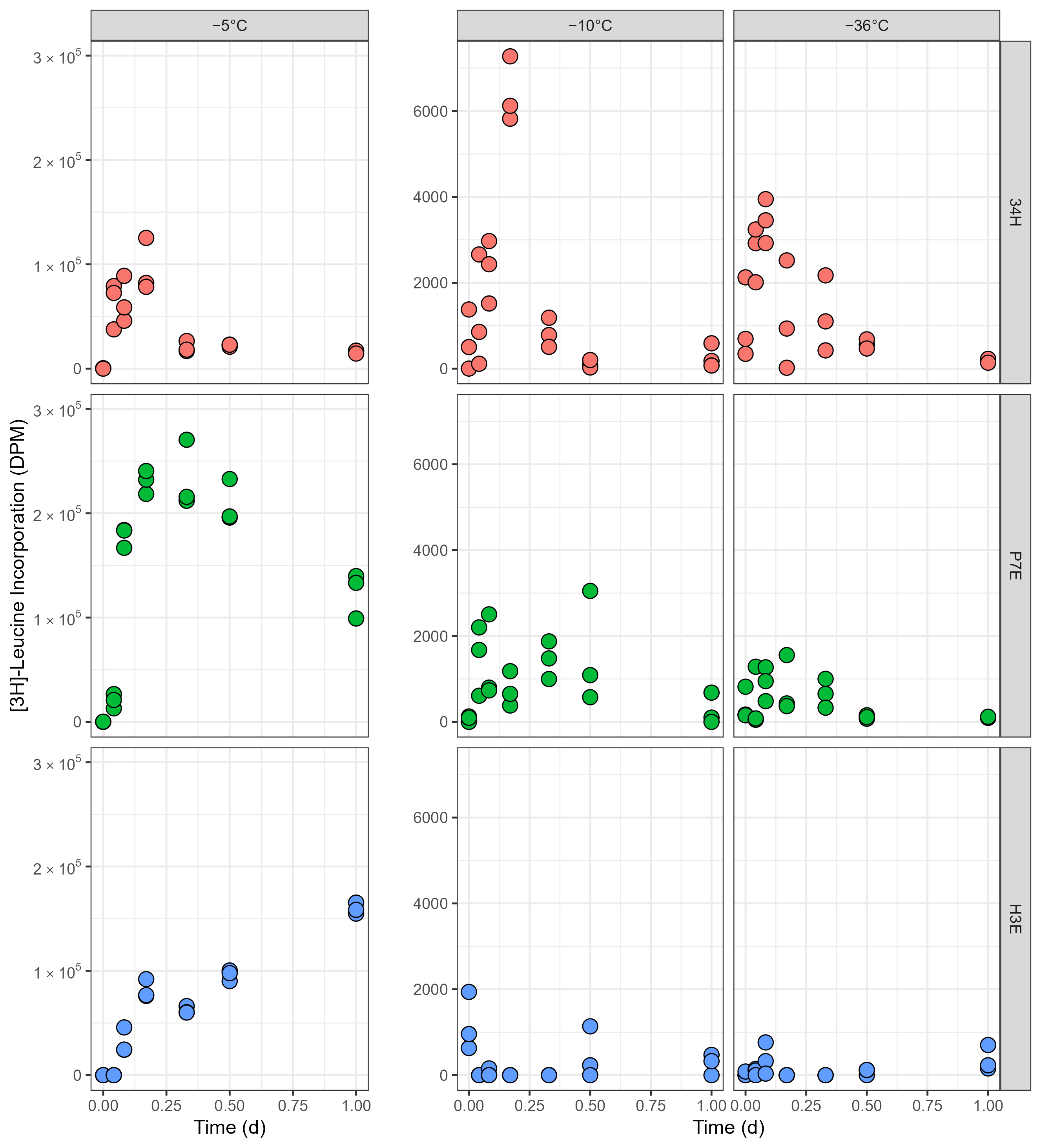

Supplement: Supplementary file 1 [file microorganisms-13-00251-s001.zip › Figure S2.png]

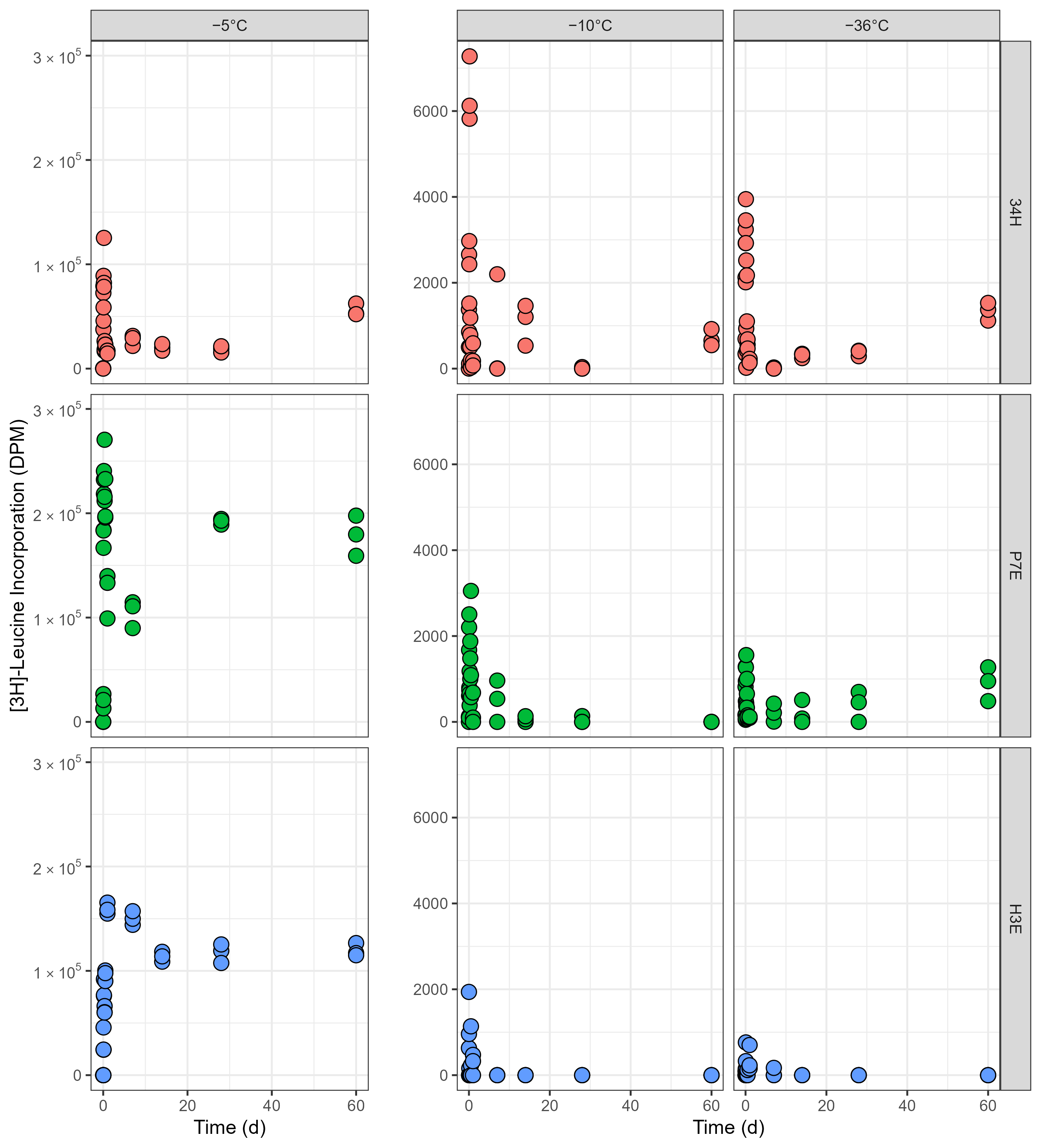

Supplement: Supplementary file 1 [file microorganisms-13-00251-s001.zip › Figure S3.png]
